# Quantifying cross-border movements and migrations for guiding the strategic planning of malaria control and elimination

**DOI:** 10.1186/1475-2875-13-169

**Published:** 2014-05-03

**Authors:** Deepa K Pindolia, Andres J Garcia, Zhuojie Huang, Timothy Fik, David L Smith, Andrew J Tatem

**Affiliations:** 1 Emerging Pathogens Institute, University of Florida, Gainesville, Florida, USA; 2 Department of Geography, University of Florida, Gainesville, Florida, USA; 3 Clinton Health Access Initiative, Boston, MA, USA; 4 Center for Infectious Disease Dynamics, Pennsylvania State University, University Park, Pennsylvania, USA; 5 Department of Biology, Pennsylvania State University, University Park, Pennsylvania, USA; 6 Department of Epidemiology, Johns Hopkins Bloomberg School of Public Health, Baltimore, USA; 7 Fogarty International Centre, National Institutes of Health, Bethesda, MD 20892, USA; 8 Department of Geography and Environment, University of Southampton, Southampton, UK

## Abstract

**Background:**

Identifying human and malaria parasite movements is important for control planning across all transmission intensities. Imported infections can reintroduce infections into areas previously free of infection, maintain ‘hotspots’ of transmission and import drug resistant strains, challenging national control programmes at a variety of temporal and spatial scales. Recent analyses based on mobile phone usage data have provided valuable insights into population and likely parasite movements within countries, but these data are restricted to sub-national analyses, leaving important cross-border movements neglected.

**Methods:**

National census data were used to analyse and model cross-border migration and movement, using East Africa as an example. ‘Hotspots’ of origin-specific immigrants from neighbouring countries were identified for Kenya, Tanzania and Uganda. Populations of origin-specific migrants were compared to distance from origin country borders and population size at destination, and regression models were developed to quantify and compare differences in migration patterns. Migration data were then combined with existing spatially-referenced malaria data to compare the relative propensity for cross-border malaria movement in the region.

**Results:**

The spatial patterns and processes for immigration were different between each origin and destination country pair. Hotspots of immigration, for example, were concentrated close to origin country borders for most immigrants to Tanzania, but for Kenya, a similar pattern was only seen for Tanzanian and Ugandan immigrants. Regression model fits also differed between specific migrant groups, with some migration patterns more dependent on population size at destination and distance travelled than others. With these differences between immigration patterns and processes, and heterogeneous transmission risk in East Africa and the surrounding region, propensities to import malaria infections also likely show substantial variations.

**Conclusion:**

This was a first attempt to quantify and model cross-border movements relevant to malaria transmission and control. With national census available worldwide, this approach can be translated to construct a cross-border human and malaria movement evidence base for other malaria endemic countries. The outcomes of this study will feed into wider efforts to quantify and model human and malaria movements in endemic regions to facilitate improved intervention planning, resource allocation and collaborative policy decisions.

## Background

Funding for malaria control has substantially increased in the past decade, reducing malaria burdens across transmission zones [1-3]. However, financial resources remain lower than the levels required to meet global eradication goals [4] and, therefore, improvements in the quantitative evidence base are important for guiding the strategic allocation of interventions. Human population movement (HPM) that leads to the movement of infections, over varying spatial and temporal scales, plays an important role in malaria dynamics across the full range of transmission intensities and epidemiological phases [5-9]. In malaria-free receptive settings (post-elimination), infection importation threatens reintroduction and resurgence [10], whilst in areas of heterogeneous risk (pre-elimination), higher transmission ‘hotspots’ may serve a infection sources (exporting infections) [5]. HPM may also lead to the emergence of drug-resistant strains of malaria that challenge control programs in both high and low transmission areas [11-13]. Quantifying both within country and cross-border movements is, therefore, important for strategic intervention planning and surveillance at a national level, and encouraging and facilitating country collaborations at a regional level.

The failure of previous elimination programmes was partly attributed to imported infections from neighbouring higher transmission risk countries [14]. In areas close to elimination, HPM from higher transmission neighbouring regions, combined with limited and unsustainable funding, continue to challenge the achievement and sustainability of malaria-free status [15]. Based on WHO recommendations, an elimination feasibility assessment conducted in Zanzibar illustrated the importance of quantifying HPM for strategic elimination planning [16,17]. Countries with higher malaria prevalence neighbours, such as the Dominican Republic, South Africa and China often exhibit higher prevalence ‘hotspots’ close to borders as a result of cross-border movements carrying infections. HPM in and out of these higher transmission regions may lead to infection flows that threaten onward transmission and burden health systems [5,7]. Drug resistance has been a major challenge among migrant groups near border areas in Asia and more recently in Africa [11]. Between-country collaborations, such as the Lumombo Malaria Control Initiative between bordering South Africa, Swaziland and Mozambique [18], and the collaborative malaria-free initiative launched in the Arabian Peninsula [19,20], were developed to tackle malaria at a regional scale. Such programmes benefit from quantitative evidence on HPM to better devise national and regional intervention and surveillance strategies [21], and refrain from repeating the inefficiencies of single-country strategies of the past [14].

In recent years, there has been a growth in the availability of data for measuring HPM across spatial and temporal scales that are important for malaria control [7]. The use of mobile phone call data records to model parasite movements, by combining HPM trajectories with malaria metric data offers one of the most promising approaches, providing fine scale estimates in space and time, and covering large percentages of national populations [5,22-24]. Analyses of mobile phone data however are constrained to within-country movements due to phone network company restrictions and do not contain information on individual-level demographics and other malaria-level characteristics, such as the use of preventive measures. Other data types, such as travel history surveys, which may contain this type of data, are restricted to small geographic areas and specific sub-populations [25]. Cross-border questionnaires remain expensive to undertake and in many malarious countries, borders are porous, with HPM through remote land border crossing points and ‘unofficial’ border points [26]. More widely used but less spatially and temporally refined are census and survey data, which contain demographic and cross-border migration data. Migration data from censuses have recently been shown to strongly correlate with movement patterns across temporal scales [27], highlighting that such data may be useful for quantifying malaria-relevant HPM. Quantitative cross-border HPM evidence has rarely been used for understanding human and malaria movements and providing guidance on extent and nature of between-country cooperation for control and elimination.

Here, to explore and illustrate the potential of census-derived migration data in quantifying cross-border human and malaria connectivities and movements, analyses of data from East Africa were undertaken. National census data for Kenya, Tanzania and Uganda were analysed to highlight patterns in cross-border migration by mapping significant origin-specific immigrant ‘hotspots’ and sub-national areas that should consider collaborating on control and elimination strategies with neighbouring countries. The data were fitted to a regression model to help explain and compare observed patterns and describe processes of immigration. Existing spatial malaria prevalence data and mathematical models were then combined with HPM data to illustrate differences in malaria movement propensities into Kenya, Tanzania and Uganda from their neighbouring regions.

## Methods

### Census data

Cross-border census migration data were obtained for Kenya, Tanzania and Uganda (Table 1). The Kenya 1999 census was obtained from the Kenya National Statistics Bureau (KNBS). Individual-level records for all individuals enumerated were available for selected variables, including current sub-location (administrative level 5 boundary) of residence, birth and previous residence location (district/administrative level 2 boundary for internal migrants and bordering country name for cross-border migrants), and demographic data on age and gender. For Tanzania, aggregated data on the number of residents in each sub-location and their nationality were obtained from the 2002 census. Demographic stratifications were not available for Tanzania. For Uganda, 2002 census micro-data, a systematic selected subset of countrywide national housing and population census data obtained from Integrated Public Use Microdata Series, International (IPUMS) [28] were obtained online. The sample contained records for all census questions for a 10% sample of all individuals enumerated. To make migration definition comparable between countries, migrants were defined based on place of birth and current residence location. The respective country censuses were also used to extract total population size per administrative boundary.

**Table 1:** Description of the type of census data available for each country

	**Kenya**	**Tanzania**	**Uganda**
**Data type**	Individual level	Aggregated data	Individual level
**Migration data**	Lifetime migration	Lifetime migration	Lifetime migration
**Additional migration data**	Recent migration	-	Recent migration
**Spatial resolution at destination**	Administrative level 5	Administrative level 5	Administrative level 2
**Spatial resolution at origin**	Administrative level 0	Administrative level 0	Administrative level 0
**Year of data collection**	1999	2002	2002
**Other variables**	Age, Gender	-	-

### Malaria data

Country-level malaria transmission maps for Kenya, Tanzania and Uganda and their respective neighbouring countries were obtained from the 2010 global *Plasmodium falciparum* endemicity maps (with *P. falciparum* parasite rate, standardized for 2-10 year olds (*Pf*PR_2-10_), for 1×1 km pixels) from the Malaria Atlas Project (MAP) [29,30]. To obtain population-weighted *Pf*PR_2-10_, an Africa-wide population distribution grid (with population density for each 1×1 km pixel) was obtained from the WorldPop Project [31] and country specific grids extracted. The endemicity maps and population grids were aligned by overlaying each country endemicity map over the population distribution grid. For each pixel on the map, population-weighted *Pf*PR_2-10_ was calculated.

### Spatial analysis

Origin-specific data on numbers of migrants (based on birth country and current residence location comparisons) were obtained for each administrative unit in the three destination countries, Kenya, Tanzania and Uganda (at different administrative resolutions, as described above). The Getis-Ord G statistic was used to estimate local ‘hotspots’ of origin-specific immigrants (based on spatial characteristics as temporal descriptions were not available for all countries). Statistically significant hotspots were determined based on a GiZScore > 1.96 (high Z scores are a measure of standard deviation associated with low p values. A GiZScore of 1.96 corresponds to p value < 0.05 and a 95% confidence interval using the standard Normal distribution assumption of theoretical spatial randomness) [32]. Significant hotspots were mapped to illustrate single-origin, as well as multiple-origin, over-lapping hotspots. Administrative units were classified into single-origin hotspots if a location was a hotspot for migrants from only one origin, and multiple-origin hotspots if a location was a hotspot for migrants from more than one origin.

### Modelling migration

Modelling migration (based on birth country and current residence location comparisons) flows can provide migration information for locations and time periods where data are not available [27]. Traditionally, human movement models have been based on the concept of gravity, that assumes a positive relationship between migrant flow and the product of population sizes as origin and destination, and a negative relationship between migrant flow and distance travelled [33,34]. To explore a possible gravity-like pattern in cross-border migration in East Africa, origin-specific immigrant occurrence was plotted against Euclidean distance from shared borders between origin and destination countries. With origins defined at a broad resolution (country level, administrative unit 0), a traditional gravity model could not be fitted. Instead, a simple positive relationship between migrant flow between origin *i*, and destination *j*, and total population size at the destination was assumed, with a negative relationship between migrant flow and Euclidean distance between origin country border and destination location for each origin-destination pair (Equation 1).

(1) $$\text{Migrant}\;\text{flo}w_{i,\;j}\;\sim \frac{\text{Total}\;\text{population}\;\text{siz}{e_{j}}^{\alpha_{i,\;j}}}{\text{Euclidean}\;\text{distanc}{e_{i,\;j}}^{{µ}_{i,\;j}}}$$

A set of 3 linear regression models (one for each destination country) were developed to quantify the variability in migrant flow as determined by destination population size and distance travelled, based on Equation 2. (Refer to Additional file 1 for expanded destination-specific equations). To achieve a linear relationship, all variables were log-transformed. To allow comparisons of the effect of destination population size and distance travelled on migrant flows between origin-destination pairs (different groups of migrants), dummy variables and interaction terms were incorporated into each model.

(2) $$\begin{array}{ll} \text{log}\left(M_{i,j}\right)= & \beta_{0}+\beta_{1}\;R_{1}+\ldots +\beta_{r}R_{r}+\alpha_{0}\text{log}\left(P_{j}\right)+\alpha_{1}R_{1}\text{log}\left(P_{j}\right)+\ldots +\alpha_{r}R_{r}\text{log}\left(P_{j}\right)+{µ}_{0}\text{log}\left(D_{i,j}\right)\\ & +{µ}_{1}R_{1}\text{log}\left(D_{i,j}\right)+\ldots +{µ}_{r}R_{r}\text{log}\left(D_{i,j}\right)+\epsilon_{i,j} \end{array}$$

*i*=*1*,…,*n*; n=number of neighbouring countries for each destination

*j*=*1*,*2*,*3*; 1=Kenya, 2=Tanzania, 3=Uganda

*M*_*i*,*j*_: Total number of migrants from origin, i, to destination, j.

*P*_*j*_: Total population size of destination location within each destination country.

*D*_*i*,*j*_: Shortest distance between origin country border and destination location.

*R*_*1*,…,*r*_: Dummy variables representing neighbouring countries per destination (r=Number of countries-1).

*β*_*0*_*,…,β*_*r*_*; α*_*0*_*,…,α*_*r*_*; μ*_*0*_*,…,μ*_*r*_: Exponents estimated from the data.

*ϵ*_*i*,*j*_: Error term.

The overall fit of the regression models was quantified using an adjusted R-squared value. The effects of population size and distance on migrant flow for each origin-specific variable were estimated by adding the origin-specific coefficient in each model to the reference variable (representing an arbitrarily chosen origin country) in each model. Significance of regression coefficients was based on p-values from t-tests to determine differences in the effect of population size and distance on migrant flow between the reference origin and all other origins for each destination.

For Kenya, this analysis was extended to include age and gender, as such information were available here. Age and gender stratified origin-specific immigrant occurrence was plotted against Euclidean distance from shared borders between origin countries and the destination country, Kenya. The regression model was extended to include age and gender as additional explanatory variables, with corresponding dummy variables created for age group and gender categories (Refer to Additional file 2 for extended equations).

### Malaria connectivities

Malaria importation propensity quantifies likely imported infection routes, as migrants are likely to maintain connections with their home locations and may engage in short term travel that, due to lost immunity, may lead to imported infections at destination. Previous analyses have shown the strong relationships between the strengths of longer-term spatial migration connectivities and shorter term movements [27]. Importation propensity estimates were based on two endemicity metrics i) mean population-weighted *Pf*PR obtained at administrative 0 level (for the entire origin country) and ii) mean *Pf*PR within a 100 km buffer from destination country border for each neighbouring origin country. *Pf*PR provides a useful measure for endemicity at large-scales, and as the migration data does not include specific origin locations, aggregated origin endemicity estimates at a national and sub-national were used. The two types of endemicity estimates for each neighbouring origin country were then multiplied by the number of origin-specific migrants in each destination country to obtain malaria importation propensity, which was relatively compared within and between the destination countries.

## Results

### Migration patterns

**Figure 1:**
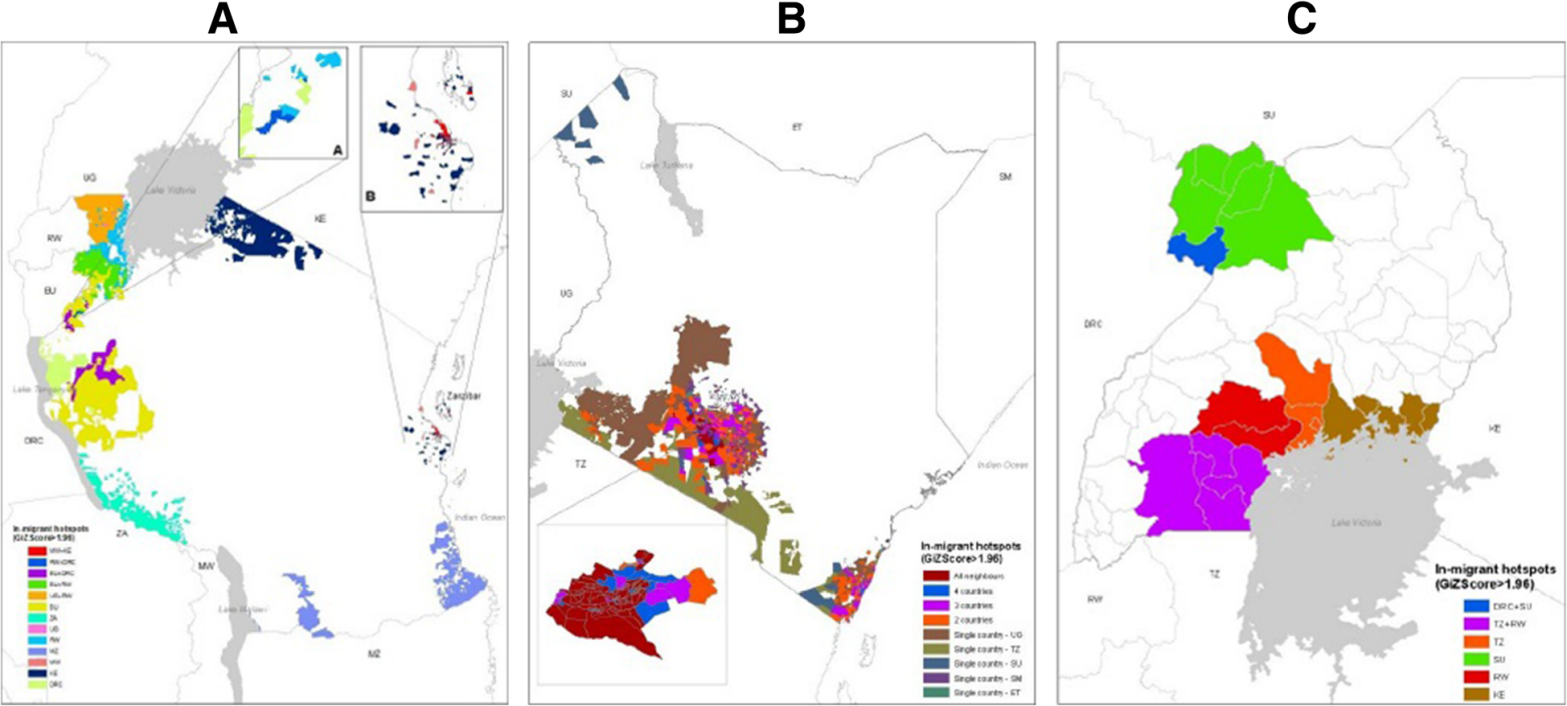
**Origin**-**specific immigrant hotspots in the three destination countries: A) Tanzania B) Kenya C) Uganda.** Statistical significance based on GiZScore > 1.96, using the Getis-Ord G statistic. Hotspots coloured based on origin country of migrants. Country codes: TZ: Tanzania, KE: Kenya, UG: Uganda, RW: Rwanda, BU: Burundi, DRC: Democratic Republic of Congo, ZA: Zambia, MW: Malawi, MZ: Mozambique, SM: Somalia, ET: Ethiopia, SU: Sudan.

Patterns of significant origin-specific immigrant hotspots differed both between and within destination countries (Figure 1). In Tanzania (Figure 1A), the highest number of origin-specific hotspots were seen close to the borders with the origin countries of the respective sets of migrants, except for migrants from Malawi, for which the majority of hotspots were near Tanzania’s capital and largest urban city, Dar es Salaam. Some locations were significant hotspots for migrants from two different countries, for example, in the northwest region, various Tanzanian sub-locations were hotspots for both Rwandan and Burundian immigrants. Near Dar es Salaam, hotspots overlapped for Kenyan and Malawi immigrants. Immigrant patterns in Kenya (Figure 1B) differed from those in Tanzania. Most distinctively, hotspot overlap was more prevalent, with some locations being quantified as hotpots for all five neighbouring countries. Additionally, Ugandan immigrant hotspots were widespread across Kenya, whilst Ethiopian and Somali immigrant hotspots were mainly in the central region of the country. Tanzanian hotspots were seen in the most populated regions across the border and near large urban centres such as Nairobi and Mombasa, whilst Sudanese hotspots were most prominent near the shared border, in the central regions around Nairobi and near Mombasa (Additional file 3). Even with the lower resolution immigrant data available for Uganda (administrative 2 level hotspots, compared to administrative 5 level hotspots in Kenya and Tanzania), overlap between origin-specific migrant hotspots was less frequent than in Kenya (Figure 1C). Nevertheless, 5 districts in southern Uganda were overlapping hotspots for migrants from both Tanzania and Rwanda, and one north-western district was a hotspot for migrants from Sudan and the Democratic Republic of Congo.

### Migration processes

**Figure 2:**
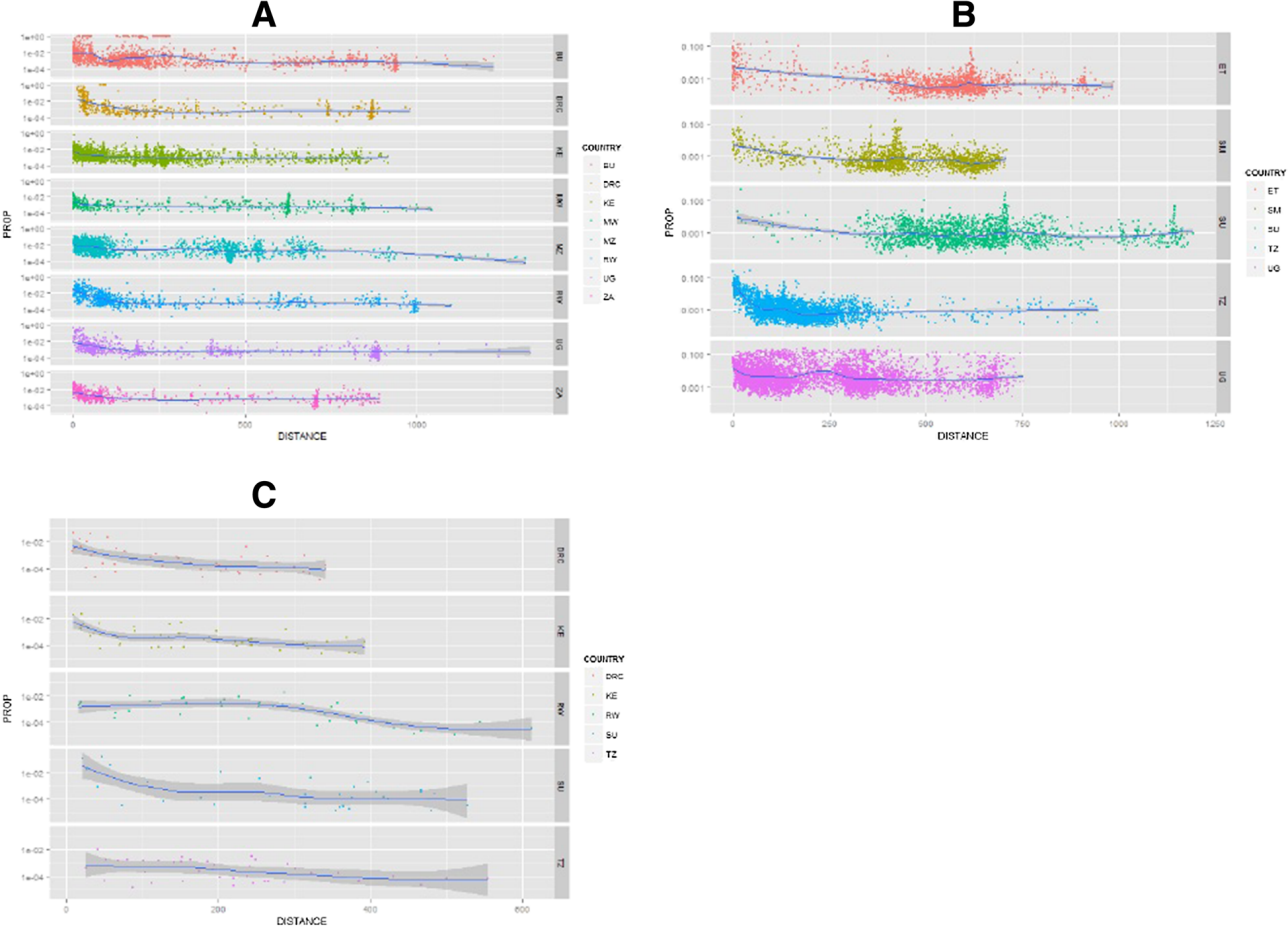
**Distance**-**migrant functions**, **illustrating the relationship between the number of migrants in each destination country: A) Tanzania B) Kenya C) Uganda, compared to the Euclidean distance from origin country border.** Y-axis is represented under a logarithmic scale to illustrate variation. Country codes: TZ: Tanzania, KE: Kenya, UG: Uganda, RW: Rwanda, BU: Burundi, DRC: Democratic Republic of Congo, ZA: Zambia, MW: Malawi, MZ: Mozambique, SM: Somalia, ET: Ethiopia, SU: Sudan.

Each origin-destination pair showed a different relationship between origin-specific immigrant abundance at the destination and the distance between the origin country border and the destination location (Figure 2). In Tanzania (Figure 2A), the largest origin-specific immigrant populations were found to be close to their respective origin country borders, illustrating an inverse relationship between immigrant population and distance between origin and destination. Clusters of immigrants were also seen in areas around and in the capital city, with the inverse relationship between distance and migrant size becoming less relevant. In Kenya, the inverse relationship between distance and migrant population abundance was mainly seen for immigrants from Tanzania (Figure 2B). Clusters close to the capital seen for Ethiopian, Somali and Sudanese migrants however were less distinct than for Tanzania, whilst Ugandans showed a more even dispersion across Kenya. Patterns were less obvious for Uganda due to the low resolution of immigrant data (Figure 2C).

Overall fits of destination-specific regression models differed, with adjusted R-square values for Kenya being 27.65%, 23.49% for Tanzania and 18.05% for Uganda. Across all origins and destinations, population size was positively associated with migration whilst distance showed an inverse relationship, except for Ugandans in Kenya (Table 2). For Tanzania and Uganda, distance was a more important determinant for migration compared to population size at destination, however in Kenya, population size at the destination location was a significant determinant for all migrant groups. For Tanzania, significant effects of distance correlated with most migrant populations being concentrated along borders, as illustrated in Figures 1 and 2. Similarly, population sizes at destination locations as a significant determinant of migration in Kenya correlated with immigration patterns illustrated in Figures 1 and 2. Within destination-specific regression models, the importance of population size and distance describing the variation in origin-specific immigrants also showed heterogeneity through differences in effects sizes. For example, in Kenya, population size had the largest effect for Somali and Sudanese migrants, compared to migrants from other origins. As Ugandan immigrants showed a more dispersed distribution (Figure 1), the effect of destination population size was the smallest compared to migrants from other origins. By including age and gender as additional explanatory variables, the model fit for Kenya improved from 27.65% to 33.14%, highlighting the importance of accounting for demographic differences [9]. Significant differences between age groups were identified for origin countries, however differences in gender remained insignificantly different throughout (Additional file 4).

**Table 2:** **Regression analysis outputs for three destination**-**specific models**, **which model migration as the dependent variable and destination population size and distance travelled as the independent variables**

		**Population size**				**Distance**			
**Model 1. destination: KE**	**Origins**	**Effect**	**se**	**t**	**p-value**	**Effect**	**se**	**t**	**p-value**
TZ^	0.32	0.01	33.23	<0.05*	-0.37	0.01	-51.55	<0.05*	
ET	0.28	0.02	-2.37	<0.05*	-0.16	0.01	16.36	<0.05*	
SM	0.40	0.02	5.43	<0.05*	-0.15	0.01	15.95	<0.05*	
SU	0.41	0.01	6.43	<0.05*	-0.09	0.03	10.89	<0.05*	
UG	0.24	0.01	-5.74	<0.05*	0.12	0.01	42.07	<0.05*	
**Model 2. Destination: TZ**	KE^	0.53	0.01	46.55	<0.05*	-0.44	0.01	-55.07	<0.05*
MZ	0.37	0.04	-4.10	<0.05*	-0.32	0.02	5.23	<0.05*	
MW	0.37	0.06	-2.48	0.01	-0.15	0.03	9.59	<0.05*	
ZA	0.23	0.06	-4.76	<0.05*	-0.34	0.03	2.94	<0.05*	
DRC	0.61	0.06	1.48	0.14	-0.75	0.05	-6.38	<0.05*	
BU	0.47	0.04	-1.56	0.12	-0.52	0.02	-3.35	<0.05*	
RW	0.45	0.06	-1.36	0.17	-0.80	0.04	-9.80	<0.05*	
UG	0.40	0.06	-2.13	<0.05*	-0.59	0.04	-3.78	<0.05*	
**Model 3. Destination: UG**	TZ^	0.40	0.01	41.44	<0.05*	-0.35	0.01	-58.89	<0.05*
KE	0.99	0.33	1.79	0.07	-1.15	0.22	-3.62	<0.05*	
SU	0.43	0.54	0.05	0.96	-1.69	0.31	-4.38	<0.05*	
DRC	1.13	0.34	2.13	<0.05*	-1.19	0.20	-4.18	<0.05*	
RW	0.77	0.35	1.04	0.30	-1.03	0.25	-2.70	<0.05*	

^reference category for each destination-specific regression model.

*significant, based on a 5% significance level.

Country codes: TZ: Tanzania, KE: Kenya, UG: Uganda, RW: Rwanda, BU: Burundi, DRC: Democratic Republic of Congo, ZA: Zambia, MW: Malawi, MZ: Mozambique, SM: Somalia, ET: Ethiopia, SU: Sudan.

Effects of each independent variable, stratified by origin country, were estimated using the regression coefficients of the interaction terms (interaction between explanatory variables and dummy variables created to represent respective origin countries).

### Malaria connectivities

**Figure 3:**
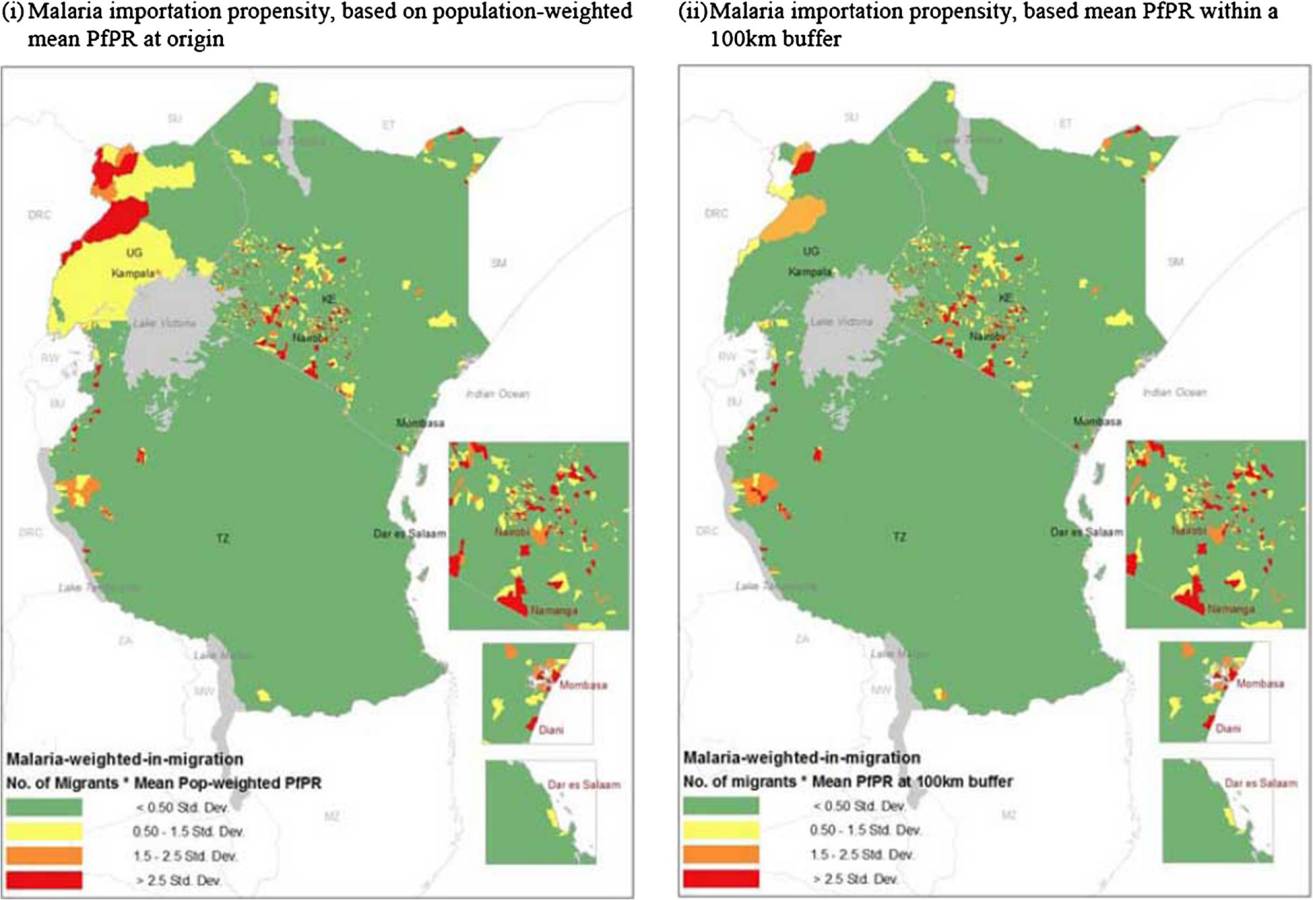
**Comparing spatial patterns of origin**-**specific propensities of malaria importation into Kenya**, **Tanzania and Uganda from neighbouring countries**, **based on two types of malaria endemicity estimate assumptions at origins (i) population**-**weighted mean *Pf*PR and (ii) mean *Pf*PR within 100 km from destination country border.** Propensity of importation = number of origin-specific migrants *origin *Pf*PR estimate. Scale represents one standard deviation from the estimated value, divided into four categories. Country codes: TZ: Tanzania, KE: Kenya, UG: Uganda, RW: Rwanda, BU: Burundi, DRC: Democratic Republic of Congo, ZA: Zambia, MW: Malawi, MZ: Mozambique, SM: Somalia, ET: Ethiopia, SU: Sudan.

**Figure 4:**
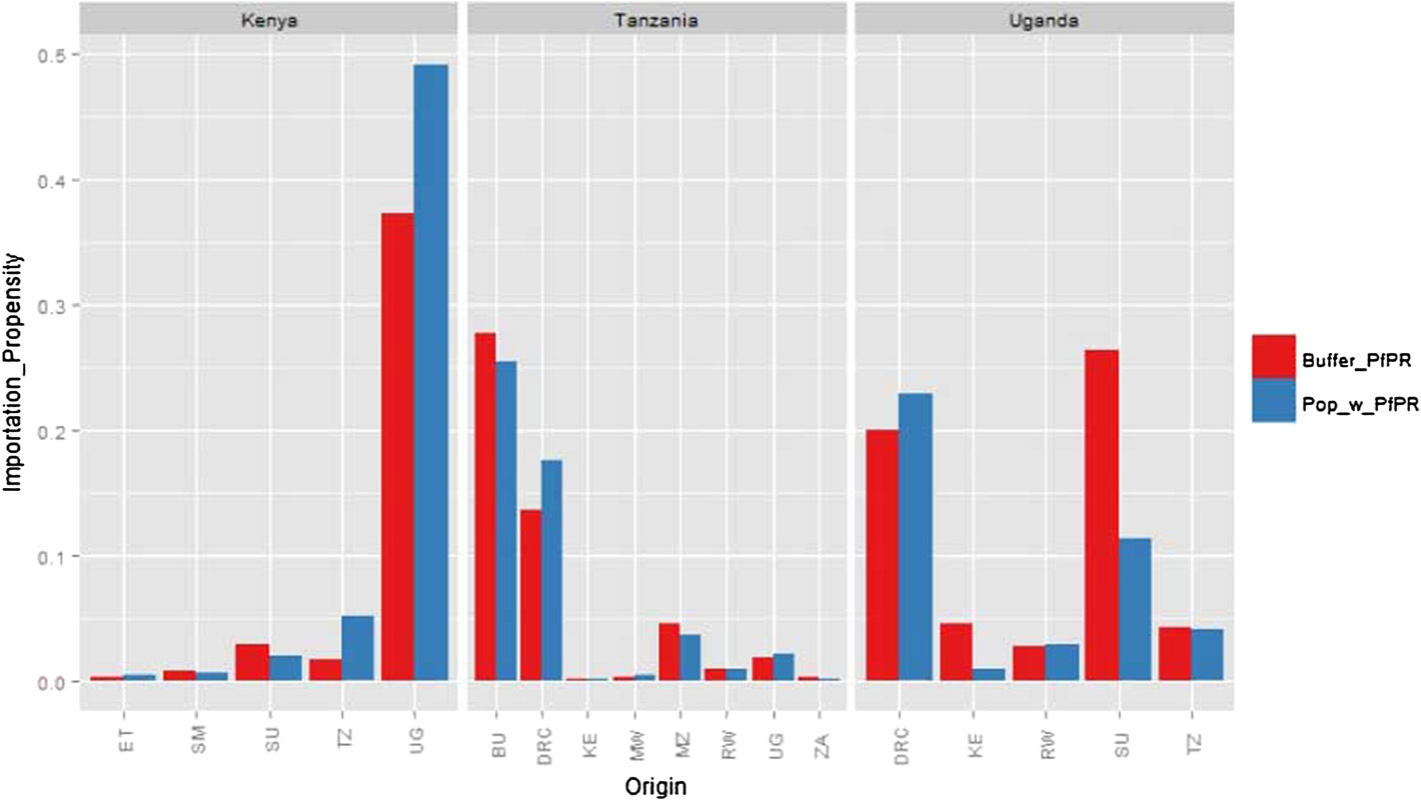
**Relative magnitudes of origin-specific malaria importation propensity into each destination country (Kenya, Tanzania and Uganda), based on two types of endemicity estimate assumptions ((i) population**-**weighted mean *Pf*PR and (ii) mean *Pf*PR within 100 km from destination country border).** The y axis shows origin-specific malaria importation propensity as a percentage of the total malaria importation propensity in the respective destination country. Country codes: TZ: Tanzania, KE: Kenya, UG: Uganda, RW: Rwanda, BU: Burundi, DRC: Democratic Republic of Congo, ZA: Zambia, MW: Malawi, MZ: Mozambique, SM: Somalia, ET: Ethiopia, SU: Sudan.

Based on the variations seen in immigrant patterns and heterogeneity in malaria transmission risk across the East African and neighbouring regions, propensities to import infections likely differs substantially between destination countries. As seen in Figure 3, under the assumptions used here, for all three East African countries, the urban areas such as Nairobi, Mombasa, Dar es Salaam and Kampala had higher estimated propensities to import infections (likely sinks of infection). The distribution of importation potential by Ugandan immigrants was widespread in Kenya, however, it was focused to north western border regions in Tanzania. Overall, based on the largest immigrant population sizes and higher endemicity in Uganda, compared to Kenya’s other neighbouring countries, propensity to import was significantly higher by Ugandans (Figure 4). In Tanzania, the largest propensities were estimated to be from immigrants from Burundi and the Democratic Republic of Congo, whilst in Uganda, estimates were largest for Sudanese and Congolese immigrants.

## Discussion

Quantifying HPM can provide useful information for evidence-based malaria control and elimination planning. As shown here, the patterns and processes of movements can differ significantly over space as well as between countries and demographic groups [9], which leads to heterogeneities in infection importation propensities, underlining the importance of accounting for local context. Quantifying these differences can aid the identification of population groups most likely to import infections, neighbouring countries and regions that are most likely to export infections (“sources”) and within country locations that are at elevated risk of importation on onward transmission (“sinks”). Identifying key population groups, sources and sinks allows national control and surveillance resources to be strategically tailored and targeted [35,36], and highlights areas and populations where further data collection studies [37] and detailed assessments can be made. As drug resistance continues to create challenges for malaria control, particularly in border regions, data on cross-border movements can inform containment strategies [35]. Moreover, quantifying these cross-border linkages and connectivities can provide indicators on when and where neighbouring countries might collaborate to plan interventions and share information at both national and regional levels (Figure 1). For example, based on the assumption that some migrant groups may have higher fluxes of travel to home countries, prophylaxis may be made available in regions where these types of migrant populations are most abundant. In East Africa, such a strategy may be adequate for DRC migrants living in Tanzania as DRC migrants are concentrated in the western regions close to borders, however would be difficult to administer for Ugandans living in Kenya, as populations are more spread across the country. In general however, the operational challenges for prophylaxis provision at this scale need consideration.

Migration patterns are heterogeneous, both within and between destination countries. Migrant flow strengths have been shown to correlate with short term movement patterns (may result due to migrants maintaining ties and visiting family at origin locations) [27], which are of importance in terms of imported infections [x], depending on endemicity levels at origins (Figures 3 and 4). Differences in the rural-urban distribution of migrant populations may therefore imply that some migrant groups may be more likely to import infections into urban areas compared to rural areas, a result that has previously been shown [9]. Due to differences in receptivity, the likelihood of onward transmission differs between rural and urban settings [38]. With heterogeneous transmission within country borders and likely significantly larger amounts of internal migration (Additional file 4), it can be important to collectively assess both internal and cross-border importation. Nevertheless, the abundance of cross-border immigrant populations provide useful indications on where countries can collaborate to develop context-specific and targeted interventions. For example, based on the migration hotspots identified here, for Kenyan malaria control strategies, it may be beneficial to highlight collaboration with neighbouring countries as a national policy, as previously done in Southern Africa [18], however, in Tanzania, collaborative work may best focused in areas close to borders.

The data and methodology used introduces some limitations into this study. Issues with census data include the difficulty of capturing up-to-date migrant trends, migrants who are fleeing from conflict or political instability [39] and other high-risk groups for infection importation, such as highly mobile populations and illegal immigrants, demonstrating the inadequacy of census data capturing the full range of HPM. Moreover, census data does not record detailed malaria-relevant characteristics, such as bed net use and access to healthcare, which would allow more detailed stratification of high-risk groups, but the integration of such datasets with georeferenced household survey information offers possibilities to overcome this [7,9]. Migration rates can be used as an indication for comparing the relative likelihoods of shorter term travel [27], however, frequencies of travel to/from home locations and elsewhere are unknown and therefore difficulties remain in estimating absolute numbers of imported infections. Furthermore, using migration as an indicator for future shorter term HPM may be less applicable for certain groups, such as those fleeing from conflict, as they are less likely to return home. Censuses generally record international migrant origins at a coarser resolution (country name) compared to within country locations (smaller administrative boundaries), making it difficult to estimate relative parasite carriage rates through malaria prevalence maps. Limitations also arise in the structure of the regression models presented here, which only include effects of distance and population size at destination on migrant flows. Other push and pull factors, such as demographics, occupation and socioeconomic factors [40], are likely to be important to include, as demonstrated here for Kenya (Additional file 2). Finally, some limitations exist in the use of *Pf*PR data as a malaria metric in this context. Mean *Pf*PR endemicity maps provide high resolution spatially-referenced metrics at large scales, but *Pf*PR is a poor measure for low transmission areas (requiring large survey samples to detect cases) [41]. Additionally, the contemporary map data used here do not provide measures of receptivity and therefore are limited in terms of assessing the effects and implications on local transmission from imported cases in an area [16].

We have presented here a framework built on census-derived migration data for providing broad assessments of cross-border human and malaria movements. While the example analyses were focused on importation to Kenya, Tanzania and Uganda, with census data widely available [42] and existing global malaria endemicity data [29], these methods can be expanded to continental scales, through the assembly of census, microdata, mobile phone call records and household surveys that record cross-border migration and HPM [7]. If movement data can be stratified by age groups and if age at which movements occur can be obtained, mathematical models can be used to estimate age-specific *Pf*PR estimates and refine estimates of propensities of groups and routes for malaria importation [43-46]. The analyses presented here represent a starting point for mobility assessments, and ideally should be supplemented with cross-border surveys [47], and other surveys with questionnaire designs that include adequate travel history questions, targeting specific mobile populations and high-risk locations. Census migration data can also be integrated with HPM estimates from mobile phone usage data and malaria surveillance data to refine importation estimates [5,22,23], though such phone data are often difficult to obtain and expensive to process, which represents a constraint for many poorly-resourced malarious regions. Through the addition of migrant characteristic descriptions, for example occupational groups and improved spatial population descriptions, more complex spatial analyses and interaction models may be utilized [48,49]. Novel analysis and modelling methods could also be developed to combine migration data with spatially-referenced drug resistance data [50] to understand migration as a determinant of drug resistance emergence [12]. Finally, with human movements playing an important role in the transmission of other diseases and a range of health concerns, the framework put forward here may also be of value in understanding epidemiological dynamics and designing intervention strategies beyond malaria.

## Conclusion

With national and international funding under threat, novel tools and techniques that improve the evidence-base for designing more efficient intervention and surveillance strategies are important. Here, a framework for utilizing existing HPM data from censuses has been developed, and combined with readily available malaria endemicity maps to illustrate how existing retrospectively gathered data can be used for quantifying cross-border movements relevant for malaria intervention and surveillance strategies. Significant variations between countries, within countries and between migrant groups were found, highlighting the importance of local context in mobility assessments and the value of such data. Identifying key regions and migrants groups enables surveillance and intervention strategies to be built around available evidence, and provides useful guidance for countries embarking on collaborative efforts.

## Competing interests

The authors declare that they have no competing interests.

## Authors’ contributions

DKP did the literature search, identified datasets, carried out the analysis and wrote the first draft of the manuscript. AJG, ZH, TF contributed to the analysis of the manuscript. DLS contributed to the analysis and review of the manuscript. AJT contributed to the writing, analysis and review of the manuscript. All authors read and approved the final version of the manuscript.

## Supplementary Material

Additional file 1 Equations for model 2 split by destination country. Click here for file

Additional file 2 Model 2 for Kenya, extending model to include age and gender. Click here for file

Additional file 3 Hotspots indicating possible between country collaborations. Click here for file

Additional file 4 **Methods used to generate networks of internal and cross-border migrants were similar to methods developed and applied in Pindolia et al [**[1]**].** With no data on cross-border migrant origins, mean in-degree and mean in-graph strength were used instead of mean degree and mean graph strength, which incorporate HPM in both directions. Click here for file
