# Supplementary material for: Quantifying cross-border movements and migrations for guiding the strategic planning of malaria control and elimination
Source: Malar J. 2014 May 3;13:169. doi: 10.1186/1475-2875-13-169 (PMC4057586; doi:10.1186/1475-2875-13-169)
Supplement: Additional file 1 — Equations for model 2 split by destination country. [file 1475-2875-13-169-S1.pdf]

Equations for model 2 split by destination country

Kenya:

$$log(M_{i,j}) = \beta_0 + \beta_1 K_1 + \beta_2 K_2 + \beta_3 K_3 + \beta_4 K_4 + \beta_5 log(P_j) + \beta_6 K_1 log(P_j) + \beta_7 K_2 log(P_j) + \beta_8 K_3 log(P_j) + \beta_9 K_4 log(P_j) + \beta_{10} log(D_{i,j}) + \beta_{11} K_1 log(D_{i,j}) + \beta_{12} K_2 log(D_{i,j}) + \beta_{13} K_3 log(D_{i,j}) + \beta_{14} K_4 log(D_{i,j}) + \varepsilon_{i,j}$$

Tanzania:

$$log(M_{i,j}) = \beta_0 + \beta_1 T_1 + \beta_2 T_2 + \beta_3 T_3 + \beta_4 T_4 + \beta_5 T_5 + \beta_6 T_6 + \beta_7 T_7 + \beta_8 log(P_j) + \beta_9 T_1 log(P_j) + \beta_{10} T_2 log(P_j) + \beta_{11} T_3 log(P_j) + \beta_{12} T_4 log(P_j) + \beta_{13} T_5 log(P_j) + \beta_{14} T_6 log(P_j) + \beta_{15} T_7 log(P_j) + \beta_{16} log(D_{i,j}) + \beta_{17} T_1 log(D_{i,j}) + \beta_{18} T_2 log(D_{i,j}) + \beta_{19} T_3 log(D_{i,j}) + \beta_{20} T_4 log(D_{i,j}) + \beta_{21} T_5 log(D_{i,j}) + \beta_{22} T_6 log(D_{i,j}) + \beta_{23} T_7 log(D_{i,j}) + \varepsilon_{i,j}$$

Uganda:

$$log(M_{i,j}) = \beta_0 + \beta_1 U_1 + \beta_2 U_2 + \beta_3 U_3 + \beta_4 U_4 + \beta_5 log(P_j) + \beta_6 U_1 log(P_j) + \beta_7 U_2 log(P_j) + \beta_8 U_3 log(P_j) + \beta_9 U_4 log(P_j) + \beta_{10} log(D_{i,j}) + \beta_{11} U_1 log(D_{i,j}) + \beta_{12} U_2 log(D_{i,j}) + \beta_{13} U_3 log(D_{i,j}) + \beta_{14} U_4 log(D_{i,j}) + \varepsilon_{i,j}$$

$M_{i,j}$: total number of migrants from origin, i, to destination, j.

P_j: total population size of destination location within each destination country.

$D_{i,j}$: Shortest distance between origin country border and destination location.

K_1: Somalia, K_2: Ethiopia, K_3: Sudan, K_4: Uganda *(do not need a dummy for the fifth country, Tanzania)*

T_1: Kenya, T_2: Uganda, T_3: Rwanda, T_4: Burundi, T_5: DRC, T_6: Zambia, T_7: Malawi *(do not a dummy for the fifth country, Mozambique)*

U_1: Kenya, U_2: Sudan, U_3: DRC, U_4: Rwanda *(do not a dummy for the fifth country, Tanzania)*

$\beta_{i,j}$ are exponents, specific to each origin-destination pair or destination, estimated from the data.
