# Supplementary material for: Quantifying cross-border movements and migrations for guiding the strategic planning of malaria control and elimination
Source: Malar J. 2014 May 3;13:169. doi: 10.1186/1475-2875-13-169 (PMC4057586; doi:10.1186/1475-2875-13-169)
Supplement: Additional file 2 — Model 2 for Kenya, extending model to include age and gender. [file 1475-2875-13-169-S2.pdf]

Model 2 for Kenya, extending model to include age and gender:

$$log(M_{i,j}) = \beta_0 + \beta_1 K_1 + \beta_2 K_2 + \beta_3 K_3 + \beta_4 K_4 + \beta_5 log(P_j) + \beta_6 K_1 log(P_j) + \beta_7 K_2 log(P_j) + \beta_8 K_3 log(P_j) + \beta_9 K_4 log(P_j) + \beta_{10} log(D_{i,j}) + \beta_{11} K_1 log(D_{i,j}) + \beta_{12} K_2 log(D_{i,j}) + \beta_{13} K_3 log(D_{i,j}) + \beta_{14} K_4 log(D_{i,j}) + \beta_{15} K_1 A_a + \beta_{16} K_2 A_a + \beta_{17} K_3 A_a + \beta_{18} K_4 A_a + \beta_{19} K_1 G_g + \beta_{20} K_2 G_g + \beta_{21} K_3 G_g + \beta_{22} K_4 G_g + \varepsilon_{i,j}$$

A_a: malaria-relevant age categories; a=8; 1: < 5 years; 2: 5-9 years; 3: 10-19 years; 4: 20-29 years; 5: 30-39 years; 6: 40-49 years; 7: 50-59 years; 8: 60 plus
G_g: gender categories; g=2; 1: male, 2: female

Regression output:

**Explanatory variables**	**Regression Coefficient**	**Standard Error**	**t**	**p-value**
**logPop**	0.3318945	0.0092155	36.015	< 2e-16
**logDist**	-0.3924936	0.0070194	-55.916	< 2e-16
**factor(AGE)2**	0.0491894	0.0365404	1.346	0.178255
**factor(AGE)3**	0.3969941	0.0317654	12.498	< 2e-16
**factor(AGE)4**	0.5879676	0.0310477	18.938	< 2e-16
**factor(AGE)5**	0.4560387	0.0317142	14.380	< 2e-16
**factor(AGE)6**	0.1964364	0.0339049	5.794	6.92e-09
**factor(AGE)7**	0.0479760	0.0380357	1.261	0.207191
**factor(AGE)8**	0.0280316	0.0389207	0.720	0.471392
**factor(GENDER)M**	0.0009421	0.0158129	0.060	0.952494
**KE_ET:logPop**	-0.0575988	0.0173310	-3.323	0.000890
**KE_SM:logPop**	0.0762631	0.0153896	4.956	7.24e-07
**KE_SU:logPop**	0.0836244	0.0142484	5.869	4.41e-09
**KE_UG:logPop**	-0.0801133	0.0122896	-6.519	7.15e-11
**KE_ET:logDist**	0.2292590	0.0124716	18.382	< 2e-16
**KE_SM:logDist**	0.2433380	0.0133431	18.237	< 2e-16
**KE_SU:logDist**	0.3116560	0.0254647	12.239	< 2e-16
**KE_UG:logDist**	0.5303973	0.0113675	46.659	< 2e-16
**KE_ET:factor(AGE)2**	-0.0038053	0.0699597	-0.054	0.956623
**KE_ET:factor(AGE)3**	-0.1433140	0.0625309	-2.292	0.021916
**KE_ET:factor(AGE)4**	-0.2848937	0.0616237	-4.623	3.79e-06
**KE_ET:factor(AGE)5**	-0.2495923	0.0646461	-3.861	0.000113
**KE_ET:factor(AGE)6**	-0.0773828	0.0701320	-1.103	0.269864
**KE_ET:factor(AGE)7**	0.0008427	0.0767247	0.011	0.991237
**KE_ET:factor(AGE)8**	0.0499196	0.0751771	0.664	0.506677
**KE_SM:factor(AGE)2**	0.0093038	0.0636394	0.146	0.883767
**KE_SM:factor(AGE)3**	-0.1397553	0.0570119	-2.451	0.014236
**KE_SM:factor(AGE)4**	-0.3212290	0.0567203	-5.663	1.49e-08
**KE_SM:factor(AGE)5**	-0.2887977	0.0581382	-4.967	6.81e-07
**KE_SM:factor(AGE)6**	-0.0198199	0.0622346	-0.318	0.750129
**KE_SM:factor(AGE)7**	0.0967190	0.0656521	1.473	0.140702
**KE_SM:factor(AGE)8**	0.1598639	0.0656984	2.433	0.014965
**KE_SU:factor(AGE)2**	0.0057587	0.0546070	0.105	0.916013
**KE_SU:factor(AGE)3**	-0.1539909	0.0489669	-3.145	0.001663
**KE_SU:factor(AGE)4**	-0.3438848	0.0491193	-7.001	2.57e-12
**KE_SU:factor(AGE)5**	-0.2580136	0.0510843	-5.051	4.42e-07

**KE_SU:factor(AGE)6**	-0.1144147	0.0542600	-2.109	0.034981
**KE_SU:factor(AGE)7**	-0.0157266	0.0600515	-0.262	0.793411
**KE_SU:factor(AGE)8**	-0.0059988	0.0591863	-0.101	0.919269
**KE_UG:factor(AGE)2**	-0.1316750	0.0458392	-2.873	0.004074
**KE_UG:factor(AGE)3**	-0.1069047	0.0416495	-2.567	0.010268
**KE_UG:factor(AGE)4**	-0.5140590	0.0413092	-12.444	< 2e-16
**KE_UG:factor(AGE)5**	-0.6951047	0.0421692	-16.484	< 2e-16
**KE_UG:factor(AGE)6**	-0.8076488	0.0443712	-18.202	< 2e-16
**KE_UG:factor(AGE)7**	-0.9628390	0.0484411	-19.876	< 2e-16
**KE_UG:factor(AGE)8**	-0.8576411	0.0491423	-17.452	< 2e-16
**KE_ET:factor(GENDER)M**	0.0442031	0.0321400	1.375	0.169036
**KE_SM:factor(GENDER)M**	0.0080601	0.0287825	0.280	0.779453
**KE_SU:factor(GENDER)M**	0.0455020	0.0258460	1.761	0.078329
**KE_UG:factor(GENDER)M**	-0.0218555	0.0213185	-1.025	0.305279

Age stratified origin-specific in-migrant occurrence plotted against Euclidean distance from shared border between origin countries and destination country, Kenya:

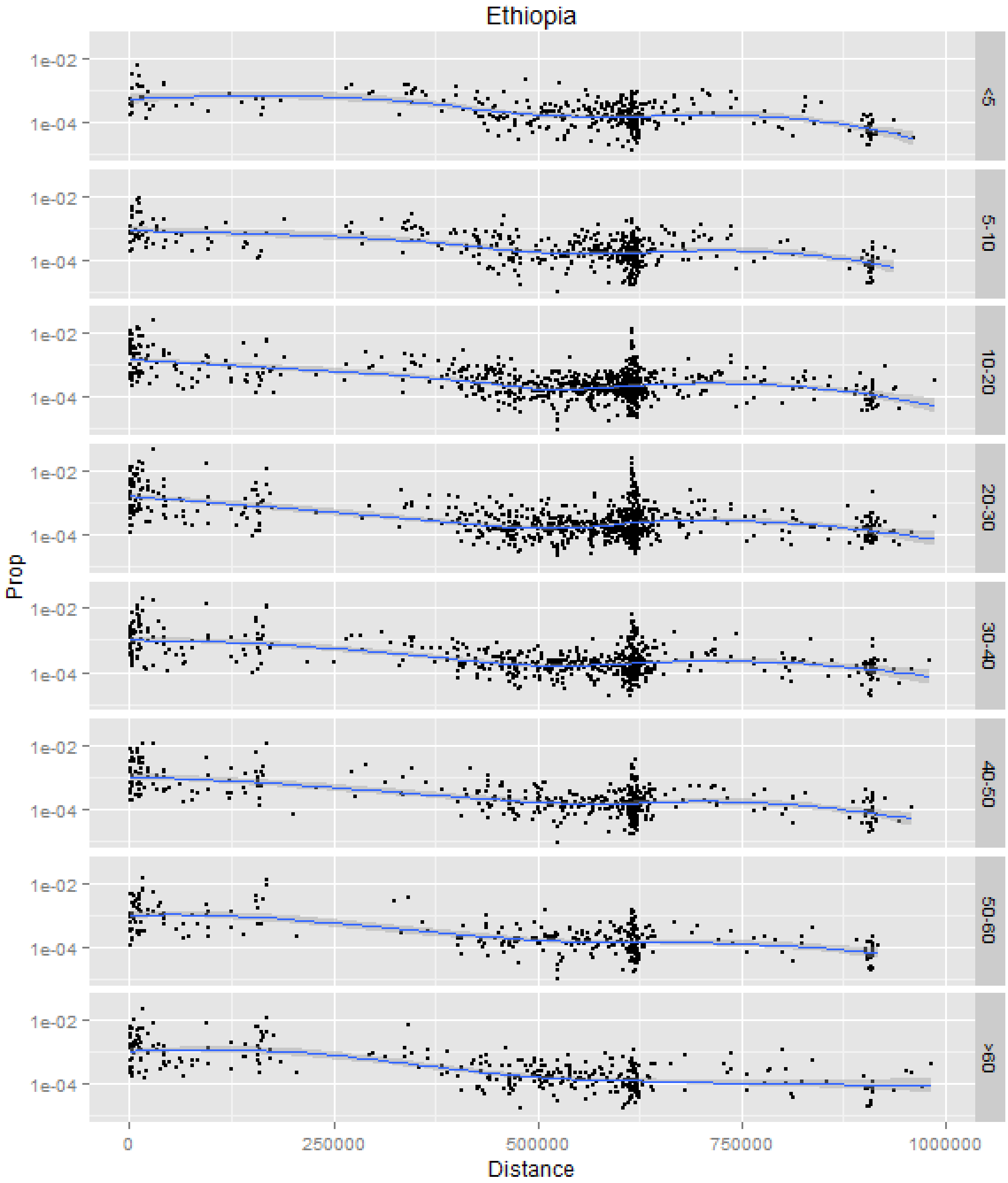

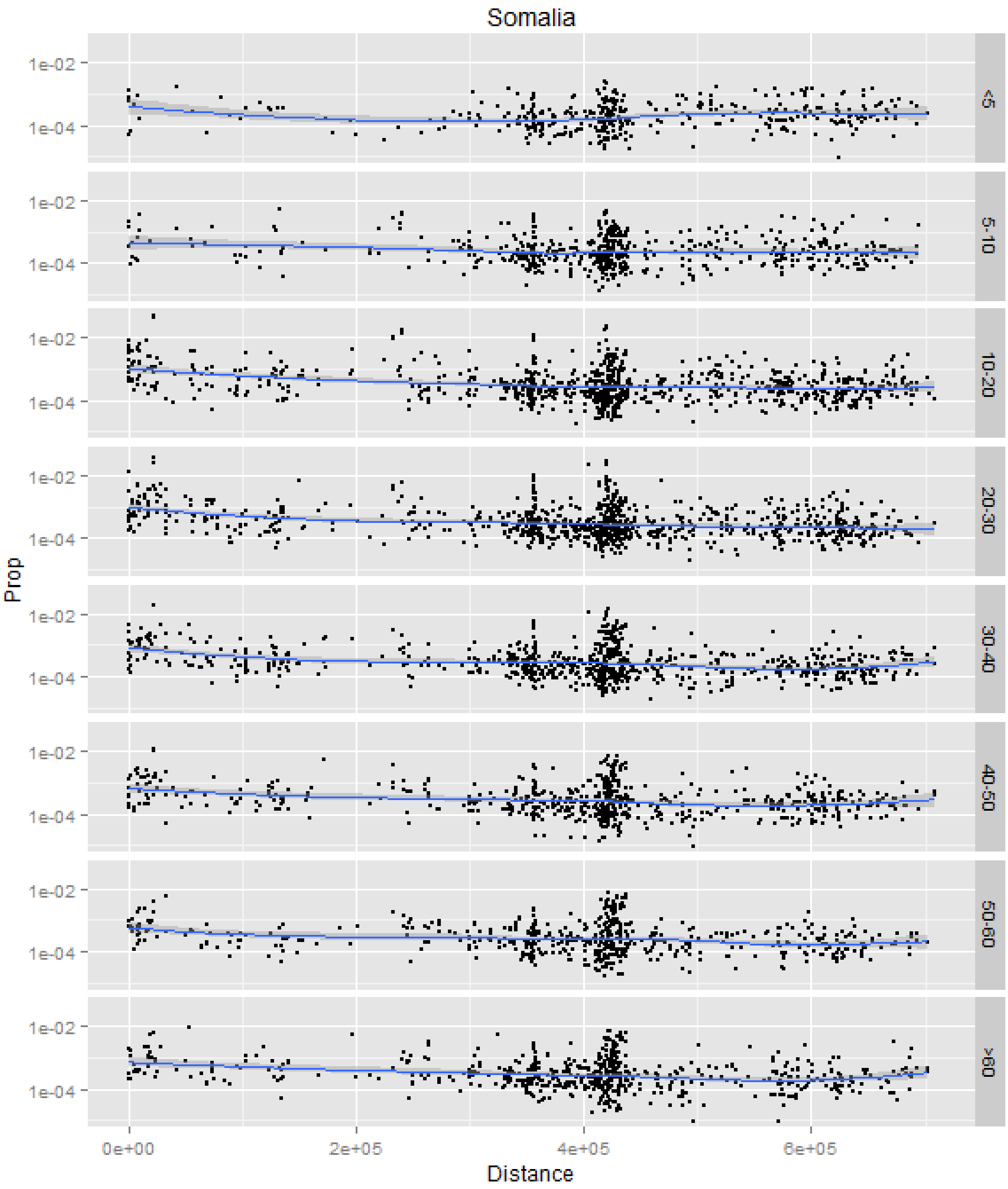
Somalia
Prop
Distance
1e-02
1e-04
<5
5-10
10-20
20-30
30-40
40-50
50-60
>60
0e+00
2e+05
4e+05
6e+05

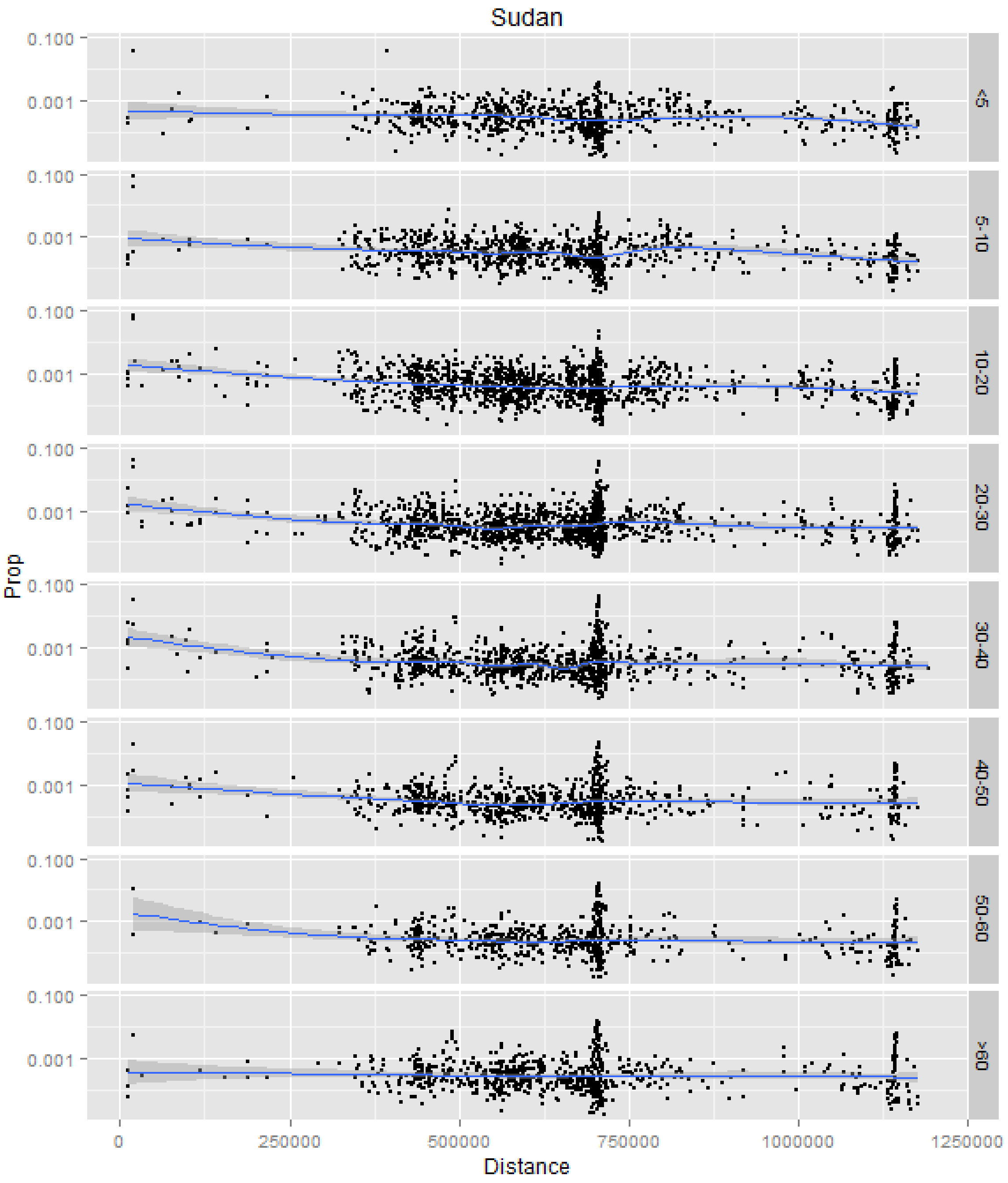

Sudan
Prop
Distance
0.100
0.001
<5
5-10
10-20
20-30
30-40
40-50
50-60
>60
0
250000
500000
750000
1000000
1250000

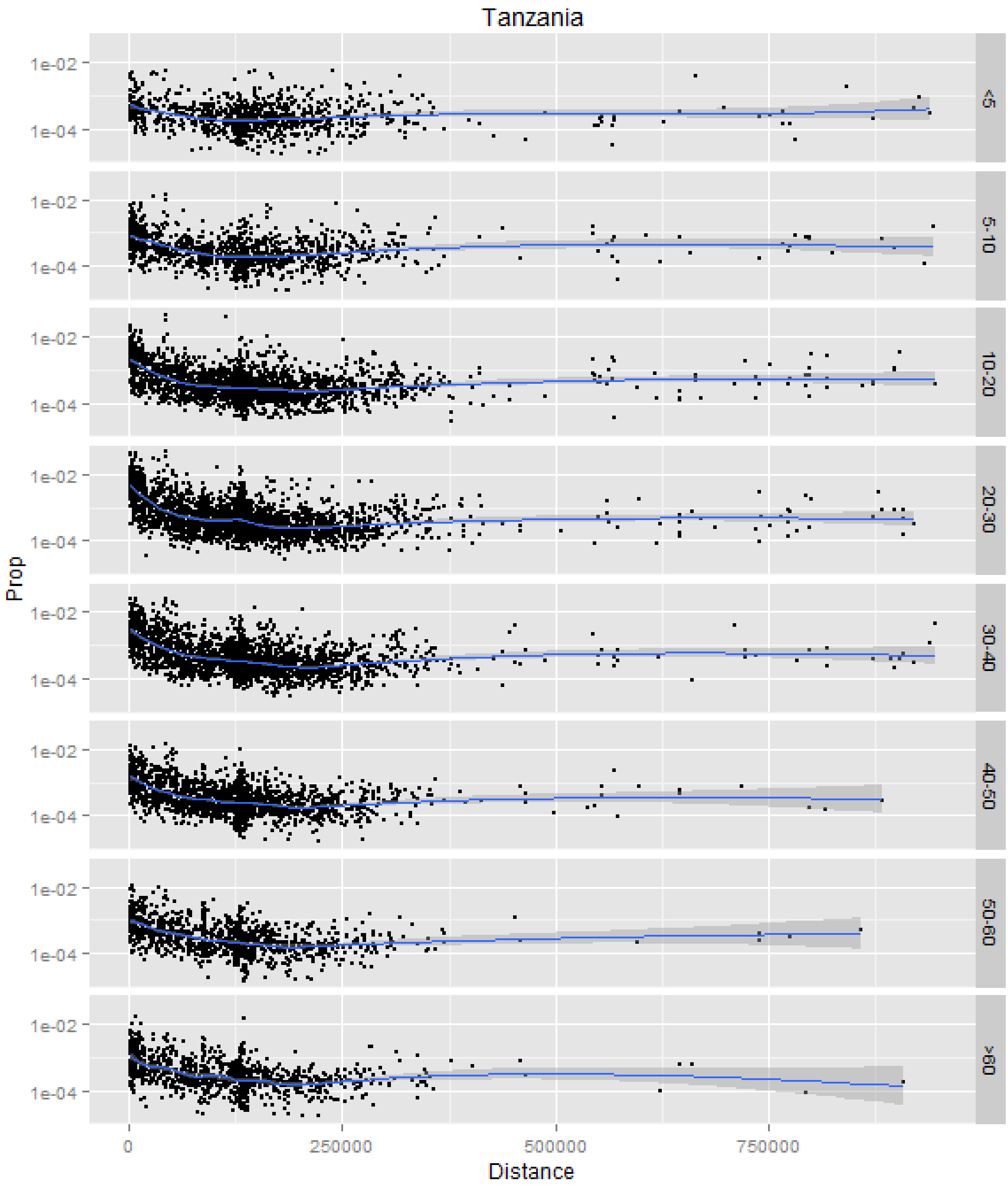
Tanzania
Prop
Distance
1e-02
1e-04
0
250000
500000
750000
<5
5-10
10-20
20-30
30-40
40-50
50-60
>60

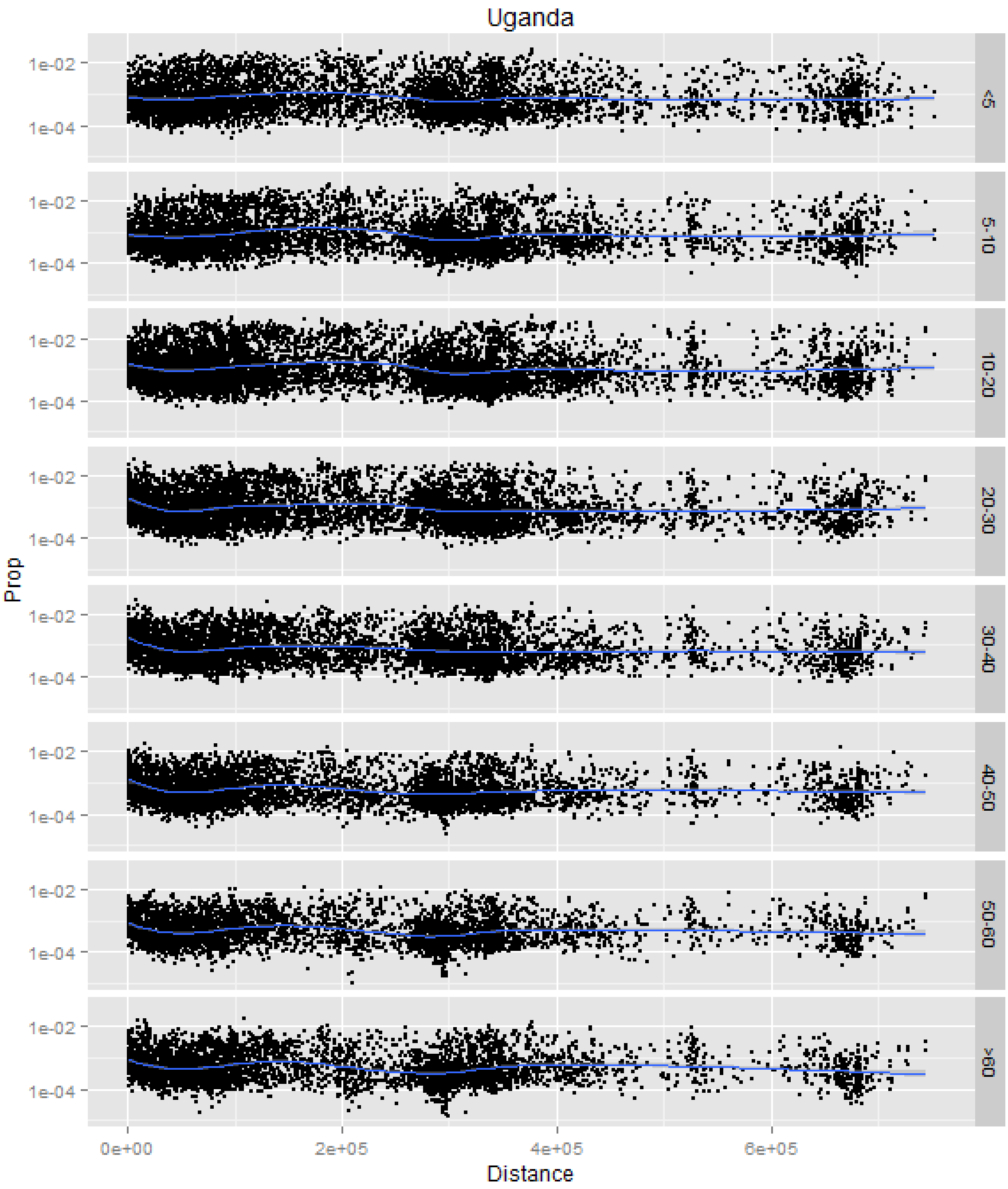

Uganda
Prop
1e-02
1e-04
<5
5-10
10-20
20-30
30-40
40-50
50-60
>60
0e+00
2e+05
4e+05
6e+05
Distance

Gender stratified origin-specific in-migrant occurrence plotted against Euclidean distance from shared border between origin countries and destination country, Kenya:

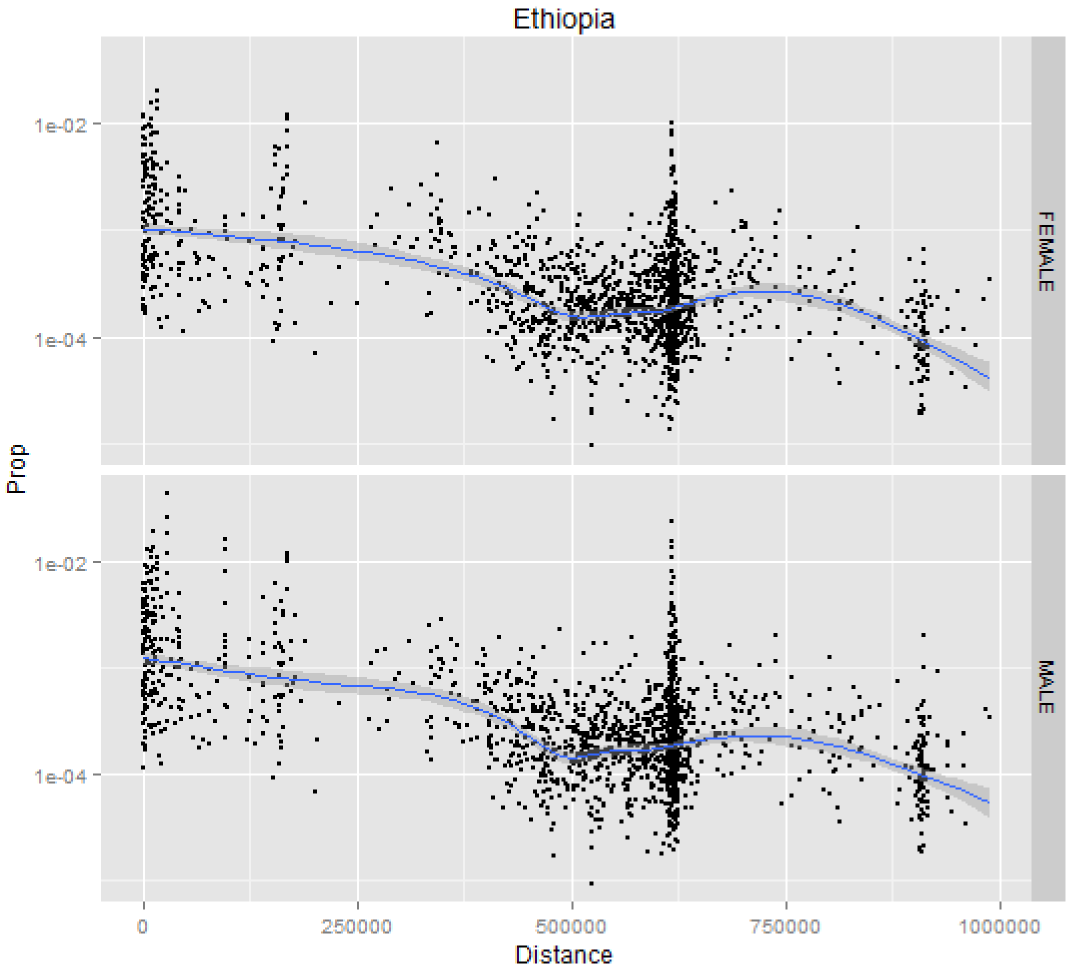

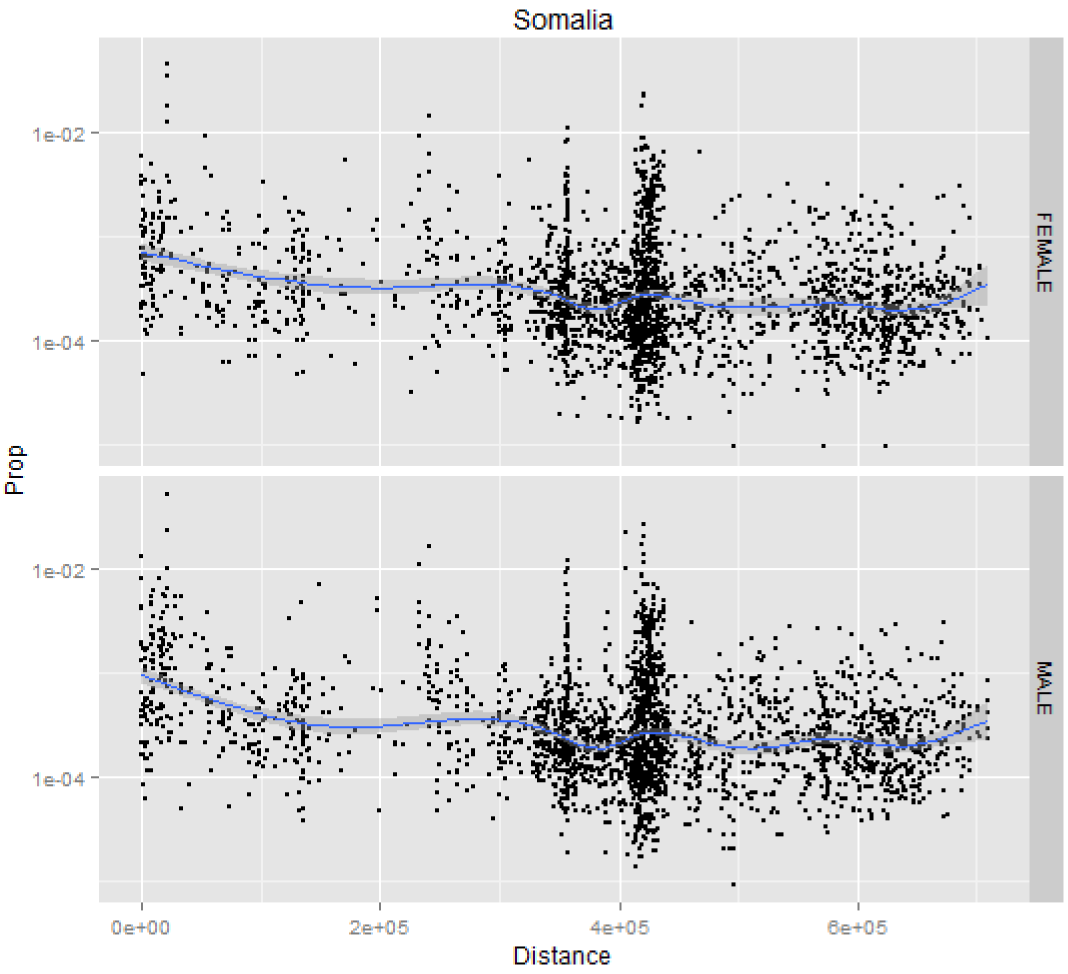

Somalia
1e-02
1e-04
FEMALE
MALE
Prop
0e+00
2e+05
4e+05
6e+05
Distance

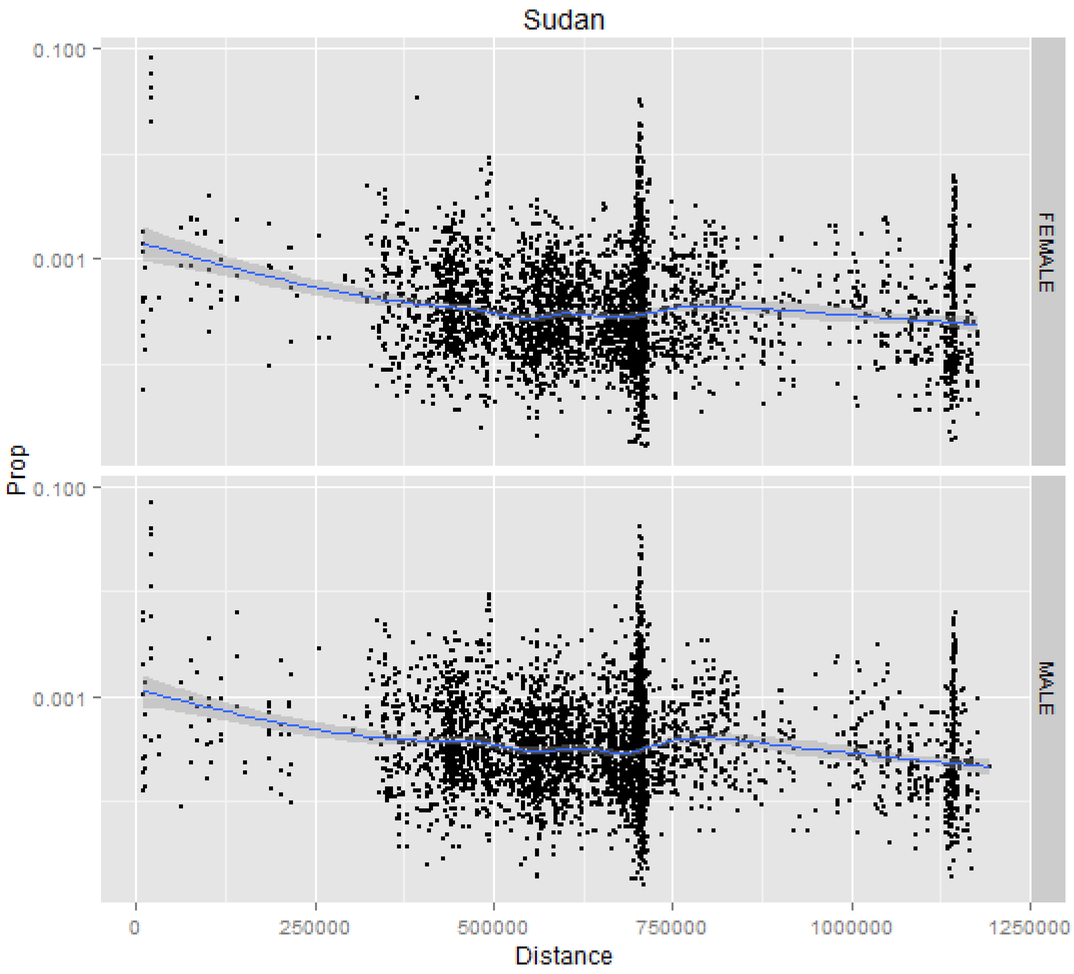

Sudan
Prop
0.100
0.001
0.100
0.001
FEMALE
MALE
0
250000
500000
750000
1000000
1250000
Distance

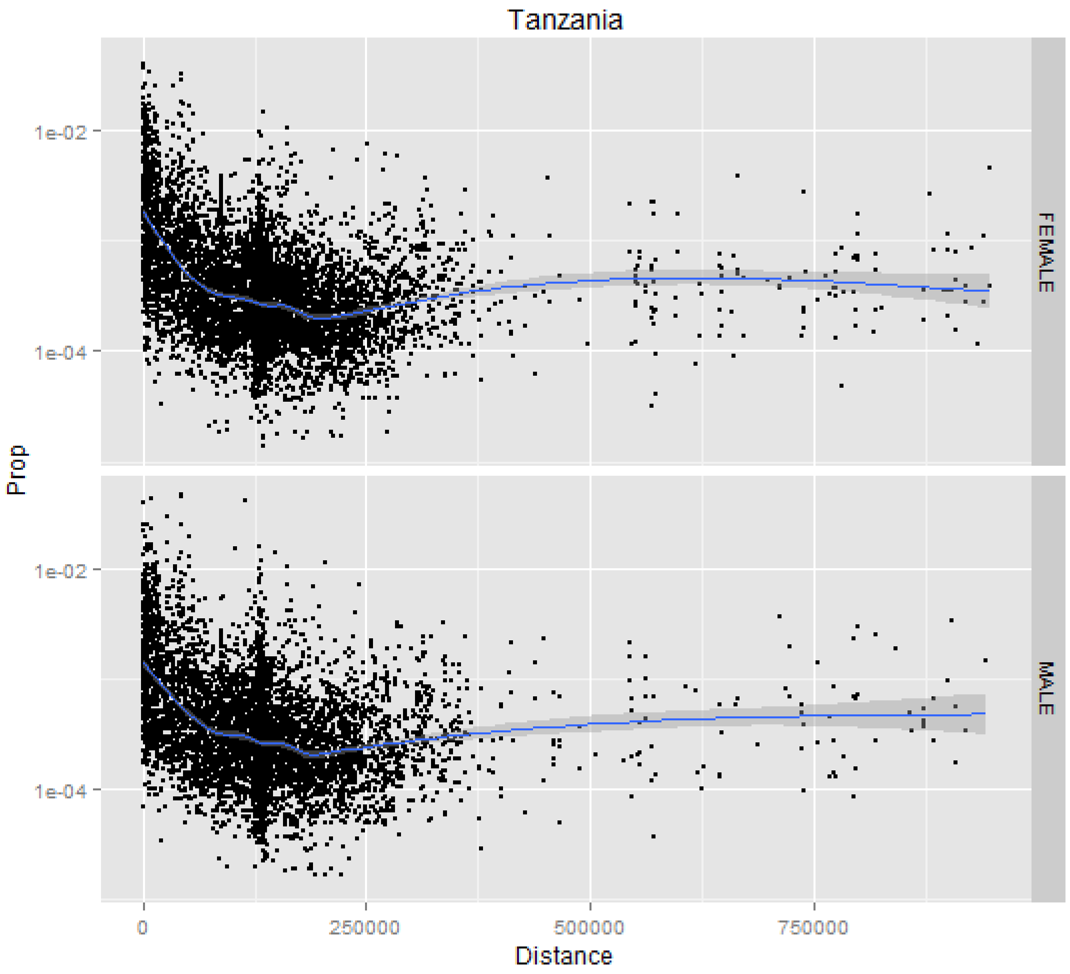
Tanzania
1e-02
1e-04
Prop
FEMALE
MALE
0
250000
500000
750000
Distance

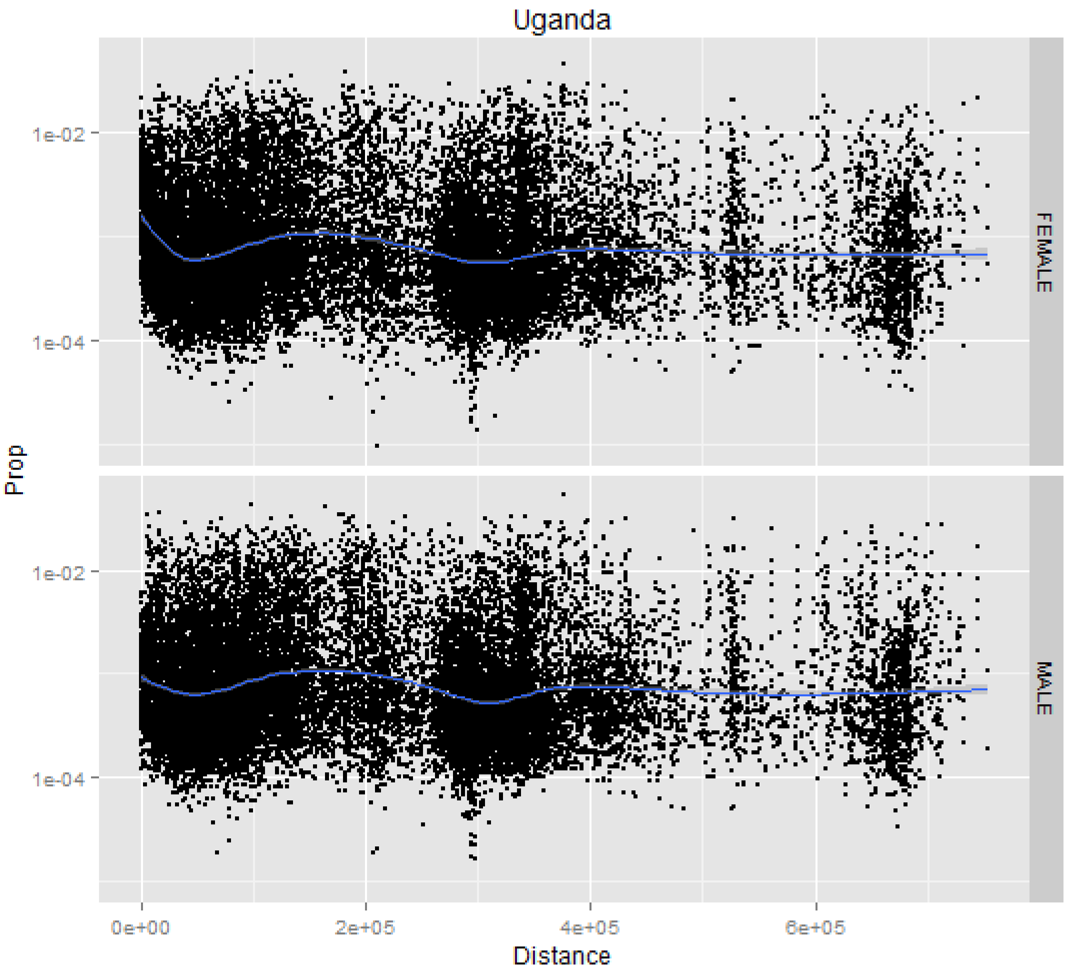
Uganda
Prop
1e-02
1e-04
FEMALE
MALE
0e+00
2e+05
4e+05
6e+05
Distance
