# Supplementary material for: Quantifying cross-border movements and migrations for guiding the strategic planning of malaria control and elimination
Source: Malar J. 2014 May 3;13:169. doi: 10.1186/1475-2875-13-169 (PMC4057586; doi:10.1186/1475-2875-13-169)

(a)Ethiopian in-migrant hotspots

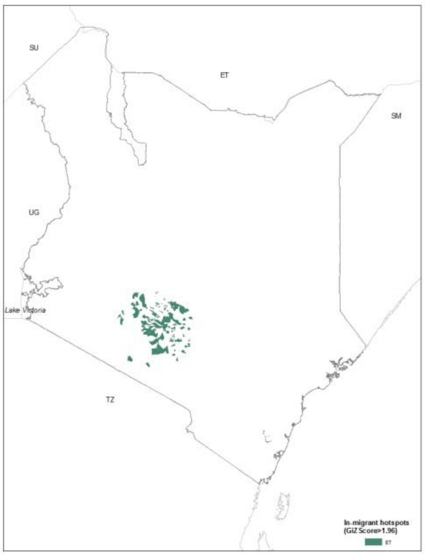

(b)Somali in-migrants

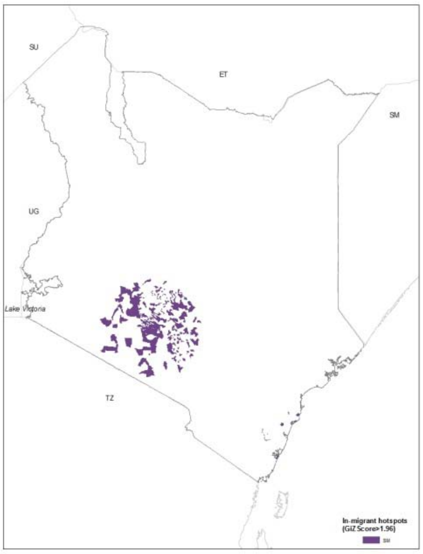

(c)Sudanese in-migrants

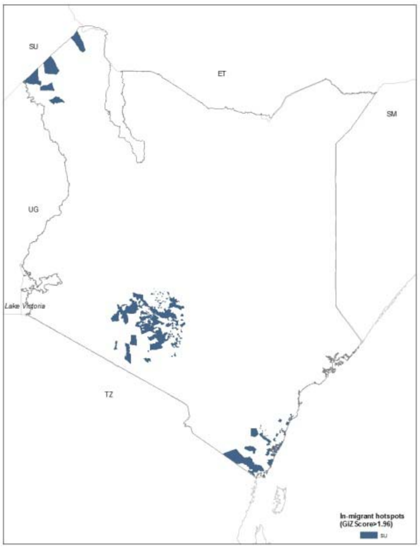

(d)Ugandan in-migrants

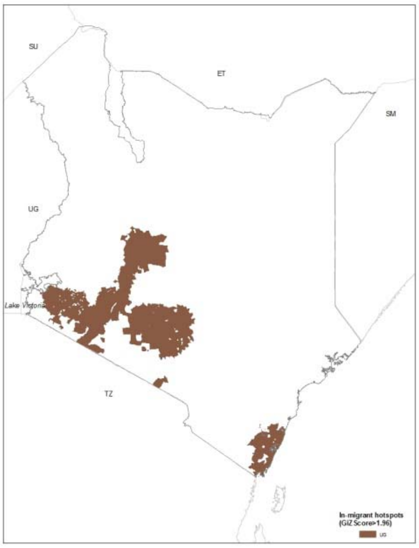

(e)Tanzanian in-migrants

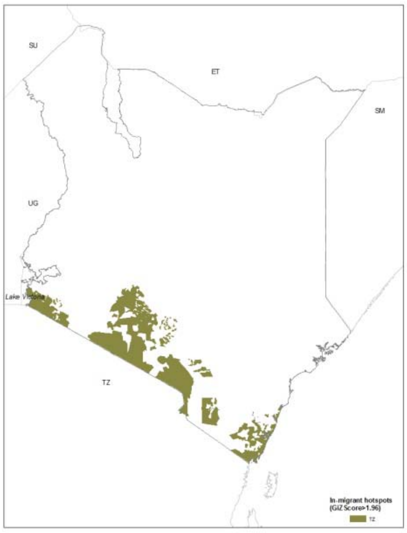

Hotspots indicating possible between country collaborations.

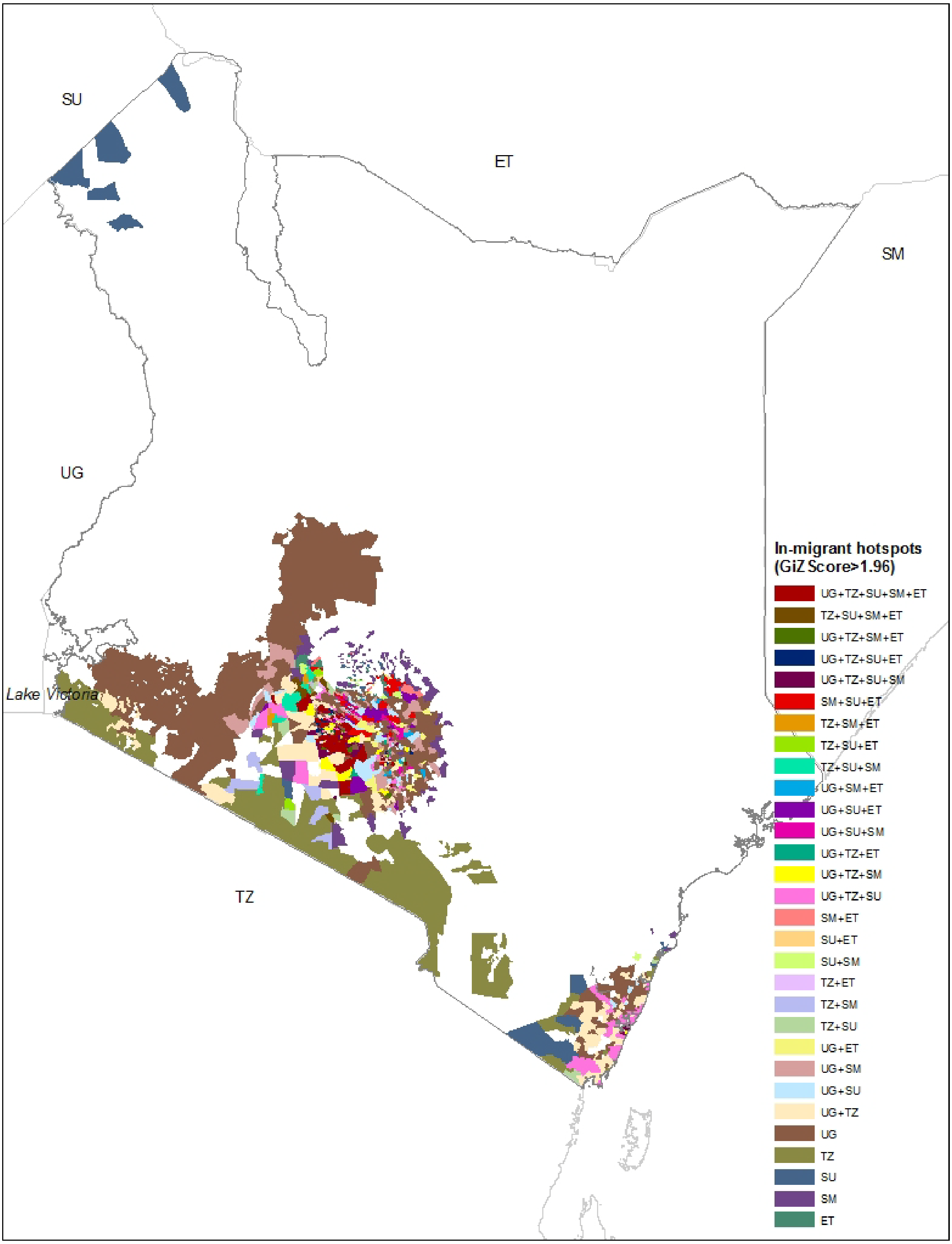

Supplement: Additional file 3 — Hotspots indicating possible between country collaborations. [file 1475-2875-13-169-S3.pdf]
