# Supplementary material for: Quantifying cross-border movements and migrations for guiding the strategic planning of malaria control and elimination
Source: Malar J. 2014 May 3;13:169. doi: 10.1186/1475-2875-13-169 (PMC4057586; doi:10.1186/1475-2875-13-169)
Supplement: Additional file 4 — Methods used to generate networks of internal and cross-border migrants were similar to methods developed and applied in Pindolia et al [[1]]. With no data on cross-border migrant origins, mean in-degree and mean in-graph strength were used instead of mean degree and mean graph strength, which incorporate HPM in both directions. [file 1475-2875-13-169-S4.pdf]

1. Comparing cross-border and within country (internal) migration in Kenya, stratified by age

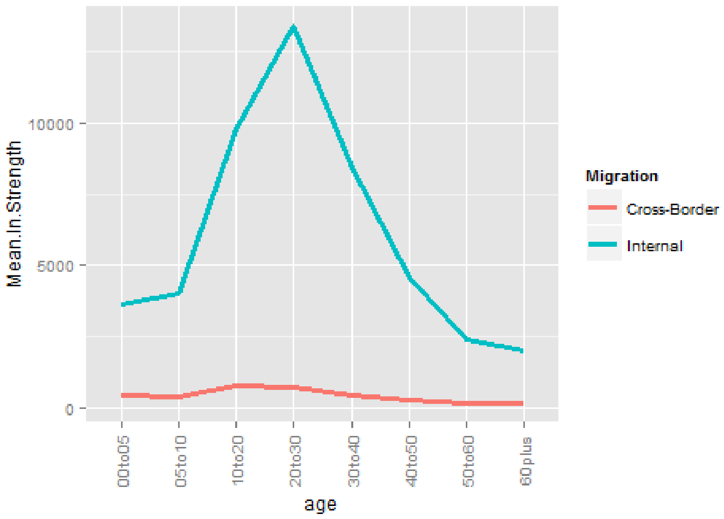

2. Comparing cross-border and within country (internal) migration in Kenya, stratified by age and gender

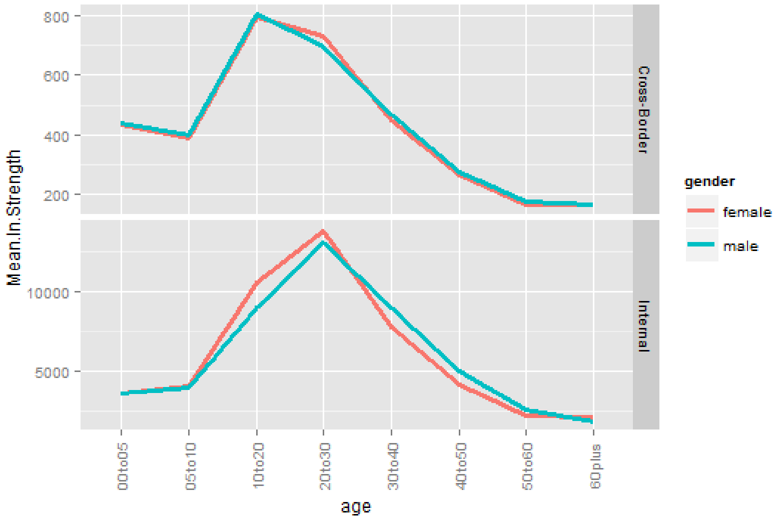

Methods used to generate networks of internal and cross-border migrants were similar to methods developed and applied in Pindolia et al [1]. With no data on cross-border migrant origins, mean in-degree and mean in-graph strength were used instead of mean degree and mean graph strength, which incorporate HPM in both directions.

References:

1. Pindolia DK, Garcia AJ, Wesolowski A, Smith DL, Buckee CO, Noor AM, Snow RW, Tatem AJ: **Human movement data for malaria control and elimination strategic planning.** *Malar J* 2012, **11:**205.
